# Assessing the risk of bias in randomized controlled trials in the field of dentistry indexed in the Lilacs (Literatura Latino-Americana e do Caribe em Ciências da Saúde) database

**DOI:** 10.1590/S1516-31802011000200006

**Published:** 2011-03-03

**Authors:** Christiane Alves Ferreira, Carlos Alfredo Salles Loureiro, Humberto Saconato, Álvaro Nagib Atallah

**Affiliations:** I MSc. Doctoral student in the field of health sciences at Universidade Federal de São Paulo — Escola Paulista de Medicina (Unifesp-EPM), São Paulo, Brazil.; II Specialist and doctoral student in the field of health sciences at Universidade Federal de São Paulo — Escola Paulista de Medicina (Unifesp-EPM), São Paulo, Brazil.; III MD, PhD. Adjunct Professor at Universidade Federal do Rio Grande Norte (UFRN), Natal, Rio Grande do Norte, Brazil.; IV MD, PhD. Titular Professor and Director of the Brazilian Cochrane Center, Universidade Federal de São Paulo — Escola Paulista de Medicina (Unifesp-EPM), São Paulo, Brazil.

**Keywords:** Randomized controlled trial [publication type], Selection bias, Double-blind method, Evidence-based dentistry, Empirical research, Ensaio clínico controlado aleatório, Viés de seleção, Método duplo-cego, Odontologia baseada em evidências, Pesquisa empírica

## Abstract

**CONTEXT AND OBJECTIVE:**

Well-conducted randomized controlled trials (RCTs) represent the highest level of evidence when the research question relates to the effect of therapeutic or preventive interventions. However, the degree of control over bias between RCTs presents great variability between studies. For this reason, with the increasing interest in and production of systematic reviews and meta-analyses, it has been necessary to develop methodology supported by empirical evidence, so as to encourage and enhance the production of valid RCTs with low risk of bias. The aim here was to conduct a methodological analysis within the field of dentistry, regarding the risk of bias in open-access RCTs available in the Lilacs (Literatura Latino-Americana e do Caribe em Ciências da Saúde) database.

**DESIGN AND SETTING:**

This was a methodology study conducted at Universidade Federal de São Paulo (Unifesp) that assessed the risk of bias in RCTs, using the following dimensions: allocation sequence generation, allocation concealment, blinding, and data on incomplete outcomes.

**RESULTS:**

Out of the 4,503 articles classified, only 10 studies (0.22%) were considered to be true RCTs and, of these, only a single study was classified as presenting low risk of bias. The items that the authors of these RCTs most frequently controlled for were blinding and data on incomplete outcomes.

**CONCLUSION:**

The effective presence of bias seriously weakened the reliability of the results from the dental studies evaluated, such that they would be of little use for clinicians and administrators as support for decision-making processes.

## Abstract (pt)

**CONTEXTO E OBJETIVO:**

Ensaios controlados randomizados (ECRs) bem conduzidos representam o mais alto nível de evidência quando a pergunta de pesquisa é sobre o efeito de intervenções terapêuticas ou preventivas. No entanto, o grau de controle de viés entre os ECRs apresenta grande variabilidade entre estudos. Por esta razão, com o aumento do interesse e produção das revisões sistemáticas e metanálises, foi necessário desenvolver metodologia suportada por evidência empírica, para incentivar e valorizar a produção de ECRs válidos e com baixo risco de viés. O objetivo deste trabalho foi realizar uma análise metodológica da área de odontologia quanto ao risco de viés de ECRs de acesso aberto, disponibilizados no banco de dados do Lilacs (Literatura Latino-Americana e do Caribe em Ciências da Saúde).

**TIPO DE ESTUDO E LOCAL:**

Trata-se de um estudo sobre metodologia conduzido na Universidade Federal de São Paulo (Unifesp) que avaliou o risco de viés dos ECRs, utilizando as seguintes dimensões: geração da sequência de alocação, sigilo da alocação, cegamento e dados sobre desfechos incompletos.

**RESULTADOS:**

Dos 4.503 artigos classificados somente 10 (0,22%) estudos foram considerados verdadeiros ECR e, destes, somente um estudo foi classificado como sendo de baixo risco de viés. Os itens mais frequentemente controlados pelos autores dos ECR foram cegamento e dados sobre desfechos incompletos.

**CONCLUSÃO:**

A presença efetiva de viés enfraqueceu seriamente a confiança nos resultados dos estudos de odontologia avaliados, sendo pouco úteis para clínicos e gestores como suporte a processos de decisão.

## INTRODUCTION

The history of the efforts to create unbiased comparison groups for preventive or therapeutic interventions goes back more than three centuries.^1^ Randomized clinical trials (RCTs), in the form in which they are known today, were introduced within medical science by Bradford Hill at the end of the 1940s. According to the Glossary of Terms in the Cochrane Collaboration, RCTs are a type of prospective study in which two or more interventions introduced by the investigator are compared. Of these, one is a control group (in which non-intervention, placebo or a standard intervention is used). RCTs use participant allocation sequences that are random.^2^

Well-conducted RCTs represent the individual highest level of evidence when the research question relates to the effect of therapeutic or preventive interventions. However, the degree of control over bias between RCTs presents great variability between studies. For this reason, with the increasing interest in and production of systematic reviews and meta-analyses, it has been necessary to develop validated tools that might help to evaluate the risk of bias in RCTs. The aim of such tools is to ensure that the clinical question is answered correctly, i.e. with an effect size that is as close as possible to the truth. RCTs with a high risk of bias may overestimate or underestimate the magnitude of interventions, and the misleading nature of such results may compromise clinical decision-making.^3^

## OBJECTIVE

The objective of the present study was to conduct a methodological analysis within the field of dentistry, regarding the risk of bias among open-access RCTs that are available in the Lilacs (Literatura Latino-Americana e do Caribe em Ciências da Saúde) database, which is a regional database covering literature from Latin America and the Caribbean.

## METHODS

The studies were retrieved through a hand search in open-access dental journals that are indexed in the Lilacs database and available on the internet (www.bireme.br). For this, a time period of six years was established (2002, 2003, 2004, 2005, 2006 and 2007).

**Figure 1.**
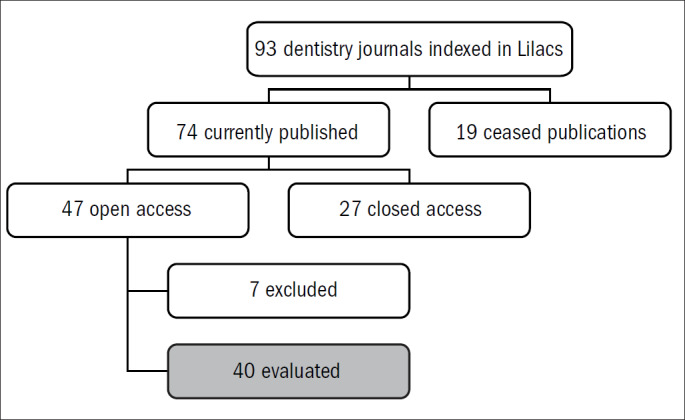
Flowchart for journal retrieval.

An initial search in Lilacs identified 93 journals within the fields of dentistry or orthodontics. Of these, 74 were current journals, 40 were open-access, seven were available on subscription and the remainder was unavailable in Lilacs. These 40 journals were selected and a hand search was conducted in these, in order to classify the study designs and identify the RCTs (Figure 1). Studies were eligible for inclusion regardless of the participants' ages, clinical conditions, contexts, genders or ethnicity. Any type of intervention carried out on human beings was eligible. Any type of outcome was eligible, and RCTs were not excluded as a function of the relevance or irrelevance of the outcomes. The main criterion for a more detailed assessment was the presence or indication of randomization or random allocation as terms in the title or abstract of study.

Empirical evidence supports at least six dimensions as potential sources of risk of bias in RCTs: randomized allocation sequence,^4^ allocation sequence concealment,^4^ blinding,^_5_^ evaluation of incomplete outcome data,^6^ selective outcome reporting^7^ and other biases. Adequate procedures for these items are important for ensuring internal validity for RCTs.^8^ The Cochrane Handbook for Systematic Reviews of Interventions recommends a tool that independently assesses these dimensions of RCTs, taking them as isolated domains without attributing weights or different ranks to them. For each of these dimensions, three response options were offered: yes (low risk of bias; score 2), when this dimension was correctly implemented and reported; no (high risk of bias; score 0), when this dimension was not implemented or not reported, or if an invalid method was reported; or unclear risk (unclear risk of bias for one or more domains; score 1), when the implementation method or the report left doubts.^2^

The classification (Higgins and Green:^2^ a classification used to summarize the risk of bias according to domains in each study and between studies) made it possible to rank each of the studies individually and to rate the validity of publications for a set of studies, as in the scheme below:

- Low risk of bias: unlikely to be enough biased to seriously change the results; within studies: low risk of bias for all domains; set of studies: most of the information comes from studies with a low risk of bias;
- Unclear risk of bias: plausible bias that raises some doubt about the results; within studies: unclear risk of bias for one or more domains; set of studies: most of the information comes from studies with a low risk of bias;
- High risk of bias: plausible bias that seriously weakens the reliability of the results; within studies: high risk of bias for one or more key domains; set of studies: the proportion of the information coming from studies with a high risk of bias is enough to affect the interpretation of the results.

To assess the risk of bias in studies considered to be potential RCTs, two independent reviewers (CAF and CAL) rated each study in order to ensure that these trials could be reproduced.^9^ Disagreements were discussed until reaching a consensus. The inter-rater reliability between the reviewers was measured using Cohen's kappa statistics. The reliability was considered outstanding: kappa = 0.824.

The statistical analyses were performed using the Statistical Package for the Social Sciences (SPSS) software, version 12.0 for Windows. Multiple logistic meta-regression was used to determine the percentage variance in the dependent variable "statistical significance" of the studies caused by the dimensions of bias. This was taken to be a yes/no category that could be explained by the following independent variables: generation of allocation, allocation sequence concealment, blinding and incomplete outcome data. Only the main outcome of the RCTs was considered, in determining the significance of the study. The method used consisted of free entry of variables and removal of variables that did not fit with the model. The significance level used in the tests was 5% (alpha = 0.05), i.e. tests were taken to be statistically significant with p values less than 5% (P < 0.05).

This study was submitted to the Research Ethics Committee of Universidade Federal de São Paulo — Escola Paulista de Medicina (Unifesp-EPM), under the number 1891/06, and was approved.

## RESULTS

Table 1 presents the frequencies and percentages of studies classified in the categories of RCT and other study designs, the frequency and percentage of unclassified studies, the absolute frequency, the annual percentage and the total number of studies evaluated.

**Table 1.** Frequencies and percentages of studies classified in the categories of randomized controlled trial (RCT) and other designs, indexed in the Lilacs database, frequency and percentage of unclassified studies, absolute frequency, annual percentage and total number of studies evaluated

Variables	Publication year						
	2002	2003	2004	2005	2006	2007	Total
Total articles	427	606	693	938	1124	1091	4879
% per year	8.80	12.40	14.20	19.20	23.00	22.40	100.00
Unclassified studies	55	64	39	54	52	34	298
Classified studies	372	542	654	884	1072	1057	4581
% unclassified	12.88	10.56	5.63	5.76	4.63	3.12	6.11
RCTs	4	15	12	8	21	18	78
Other designs	368	527	642	876	1051	1039	4503
% RCTs	1.09	2.85	1.87	0.91	2.00	1.73	1.73

Lilacs = Literatura Latino-Americana e do Caribe em Ciências da Saúde.

The journals evaluated contributed 4,879 articles. Among these, 4,581 could be classified regarding the study design, while no classification was possible for 298 (6.11%). Among the classified studies, 78 (1.73%) were initially classified as potential RCTs, while 4,503 were classified as other types of design. Over the study period, the data indicated a trend of growth in the total number of studies and in the number of RCTs.

Table 2 presents the studies, evaluated according to author, year of publication, classification of risk of bias in the dimensions, classification of the risk of bias in each study, the statistical significance of the main outcome assessed and the frequency of dimensions with a low risk of bias in each study.^10–87^

**Table 2.** Authors, year of publication, methodological characteristics and classification of risk of bias in each potential RCT, statistical significance of the main outcome assessed and the frequency of dimensions with a low risk of bias

Authors	Year	Sequence generation	Allocation sequence concealment	Blinding	Incomplete outcome data	Risk of bias	Significance	Dimensions with low risk of bias
Aguiar and Saliba^10^	2004	No	No	Yes	Yes	High	Yes	2
Alves et al.^11^	2003	No	No	Yes	Unclear	High	No	1
Amaral et al.^12^	2006	No	No	No	Unclear	High	Yes	0
Andrade et al.^13^	2005	No	No	Yes	Unclear	High	No	1
Bellón Leyva and Calzadilla Mesa^14^	2007	No	No	No	Unclear	High	Yes	0
Bermudez-Cuesta et al.^15^	2004	No	No	No	Unclear	High	Yes	0
Bortolluzi et al.^16^	2007	No	Yes	Yes	Yes	High	No	3
Botelho et al.^17^	2007	Yes	No	Yes	Yes	High	No	3
Braga et al.^18^	2006	No	No	No	Yes	High	Yes	1
Calvo et al.^19^	2006	No	No	Yes	Yes	High	No	2
Camps Mullines et al.^20^	2007	No	No	No	Unclear	High	No	0
Cardoso and Buzalaf^21^	2004	No	No	No	Unclear	High	No	0
Castillo Castillo et al.^22^	2006	No	No	No	Yes	High	Yes	1
Chambrone et al.^23^	2007	No	No	Yes	Unclear	High	No	1
Colombo et al.^24^	2003	Yes	No	Yes	Unclear	High	Yes	1
Conti et al.^25^	2005	No	No	Yes	Unclear	High	Yes	1
Corona Carpio et al.^26^	2006	No	No	No	No	High	NA	0
Cortés Vargas et al.^27^	2002	No	No	Yes	Yes	High	No	2
Costa et al.^28^	2007	No	Yes	Yes	Yes	High	No	3
Cruz Chu and Díaz-Pizán^29^	2005	No	No	No	Unclear	High	No	0
Cury et al.^30^	2003	No	No	Yes	Unclear	High	No	1
Damante et al.^31^	2004	No	No	Yes	Unclear	High	No	1
de Assis et al.^32^	2006	Yes	No	Yes	Yes	High	No	3
Dercelli et al.^33^	2007	No	No	Yes	Unclear	High	No	1
Dourado et al.^34^	2005	No	No	No	Unclear	High	No	0
Elias et al.^35^	2006	No	Unclear	No	No	High	No	0
Faraco et al.^36^	2003	Unclear	No	Yes	Unclear	High	No	1
Frazao et al.^37^	2004	No	Unclear	Yes	Yes	High	No	2
García López et al.^38^	2003	No	No	No	No	High	Yes	0
García Arocha et al.^39^	2004	Yes	No	Yes	Yes	High	No	3
Gentille and Greghi^40^	2004	No	No	No	Unclear	High	No	0
Gispert Abreu et al.^41^	2004	Unclear	No	Unclear	Yes	High	Yes	1
Gispert Abreu et al.^42^	2006	No	No	Unclear	Yes	High	Yes	1
Gómez et al.^43^	2003	No	No	Unclear	Yes	High	No	1
Gonçalves et al.^44^	2004	Yes	No	Yes	Unclear	High	Yes	2
Gonzáles Rodríguez et al.^45^	2007	No	No	No	Unclear	High	Yes	0
Grisi et al.^46^	2006	No	No	Yes	No	High	No	1
Guimarães et al.^47^	2006	No	Yes	Yes	Yes	High	No	3
Kogawa et al.^48^	2005	Yes	No	Yes	Unclear	High	Yes	2
Laureano Filho et al.^49^	2003	No	No	No	Unclear	High	No	0
Lima et al.^50^	2003	No	Yes	Yes	No	High	No	2
Llerena et al.^51^	2006	No	No	Unclear	Unclear	High	NA	0
Martínez Ramos et al.^52^	2007	No	No	No	No	High	NA	0
Medina Vega et al.^53^	2006	No	No	No	Unclear	High	No	0
Miura et al.^54^	2007	No	No	No	Yes	High	No	1
Mora Loya et al.^55^	2003	Yes	No	No	Unclear	High	No	1
Moraes et al.^56^	2007	No	Unclear	No	Unclear	High	NA	0
Orellana et al.^57^	2007	No	No	Unclear	Unclear	High	No	0
Ozaki et al.^58^	2006	Yes	Yes	Yes	Yes	Low	No	4
Pannuti et al.^59^	2003	No	Unclear	Unclear	Yes	High	No	1
Pannuti et al.^60^	2003	No	No	Unclear	Unclear	High	Yes	0
Peña Ruiz et al.^61^	2007	No	No	Unclear	Yes	High	No	1
Pereira et al.^62^	2007	No	No	No	Unclear	High	Yes	0
Peruzzo et al.^63^	2007	Yes	Yes	No	Unclear	High	Yes	2
Pinheiro et al.^64^	2004	No	No	No	No	High	No	0
Proaño de Casalino and Guillen Galarza^65^	2005	Yes	No	No	Unclear	High	Yes	1
Querido et al.^66^	2004	No	No	Unclear	Unclear	High	NA	0
do Rego et al.^67^	2003	No	No	No	Unclear	High	Yes	0
Rivera et al.^68^	2006	No	No	Yes	Unclear	High	NA	1
Rojano Santillán et al.^69^	2004	No	No	No	Unclear	High	No	0
Rojas de Morales et al.^70^	2002	Unclear	No	Unclear	No	High	Yes	0
Roscher et al.^71^	2004	No	No	No	Yes	High	No	1
Salazar de Plaza et al.^72^	2002	No	No	No	No	High	NA	0
Salgado et al.^73^	2006	No	Yes	Unclear	Yes	High	Yes	2
Samra Quintero et al.^74^	2006	No	No	No	Unclear	High	No	0
Shintome et al.^75^	2007	No	No	No	Unclear	High	Yes	0
Silva et al.^76^	2006	No	No	No	Unclear	High	Yes	0
Soares et al.^77^	2006	No	No	No	Unclear	High	NA	0
Tavares et al.^78^	2007	No	No	No	No	High	NA	0
Torres et al.^79^	2007	No	No	No	Yes	High	No	1
Ubeda et al.^80^	2005	No	No	Unclear	Unclear	High	Yes	1
Valle García et al.^81^	2002	No	No	No	Unclear	High	Yes	0
Vasconcelos et al.^82^	2005	No	No	No	Unclear	High	No	0
Vasconcelos et al.^83^	2006	No	No	No	Unclear	High	Yes	0
Vergani et al.^84^	2004	No	No	Yes	No	High	No	1
Vieira et al.^85^	2006	No	No	Unclear	Yes	High	Yes	1
Virga et al.^86^	2006	No	No	Unclear	No	High	Yes	0
Zanata et al.^87^	2003	Unclear	No	No	No	High	Yes	0
**RCT Low risk of bias**		**10**	**7**	**26**	23	**1**		**66**

NA = not assessed; RCT = randomized controlled trial.

Among the 78 studies evaluated, 77 (98.72%) were classified as presenting a high risk of bias for all dimensions assessed; 6 (7.69%) for three dimensions; nine (11.54%) for two dimensions; and 27 (34.62%) for only one dimension. A single study (1.28%)^58^ was classified as presenting low risk of bias for all of the dimensions. In summary, 77 (98.72%) of the articles were classified as studies presenting a high risk of bias and only one study (1.28%) was classified as presenting a low risk of bias. None of the studies was classified as presenting an unclear risk of bias, since the combination of one unclear dimension and other dimensions with a low risk of bias did not occur.

With regard to statistical significance, 28 studies (35.90%) presented an effect size with a significant difference in favor of the intervention, while there was no significant difference in 40 studies (51.28%) and nine studies (11.54%) were not formally assessed, statistically. All of the studies that controlled for three or more dimensions presented non-significant results.

**Figure 2.**
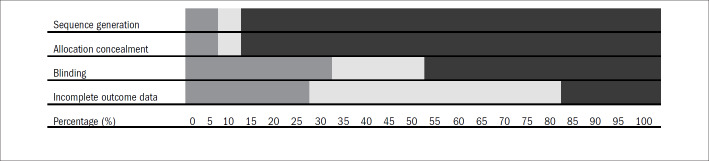
Risk of bias shown graphically, between high, unclear and low risk, for the four dimensions evaluated in the 78 open-access randomized controlled trials (RCTs).

Legend: grey = low risk of bias; white = unclear risk of bias; black = high risk of bias

Figure 2 presents the risk classification graphically, between high, unclear and low risk, for the four dimensions evaluated in the 78 open-access RCTs evaluated.

Among the 78 studies evaluated, the dimension for which the risk of bias was most correctly controlled for was blinding, in which 26 studies (33.33%) presented a low risk of bias. This was followed by controlling for incomplete outcome data, in which 23 studies (29.49%) were classified as presenting a low risk of bias. Ten studies (12.82%) presented a low risk of bias in relation to the method for generating the allocation, seven studies (8.97%) in relation to allocation sequence concealment. Thirty-six studies (46.15%) among the sample of studies evaluated did not control for the risk of bias relating to any of the dimensions under examination.

Table 3 presents the results from the logistic meta-regression for evaluating the association between the risk of bias in the domains assessed and the statistical significance of the main outcomes of the studies.

**Table 3.** Logistic meta-regression, risk of bias in the domains assessed and the statistical significance of the main outcomes of the studies

Variable	B	Standard error	Wald's test	df	Sig	Exp(B)	95% CI	
							Lower boundary	Upper boundary
Sequence generation	0.706	0.392	3.238	1	0.072	2.025	0.939	4.368
Blinding	−0.826	0.327	6.400	1	0.011	0.438	0.231	0.830
Constant	0.157	0.371	0.179	1	0.673	1.170		

Variables used in the first stage: sequence generation, allocation concealment, blinding and incomplete outcome data.

Dependent variable: significant difference.

CI = confidence interval; df = degrees of freedom; Sig = significance; Exp(B) = beta exponent.

The logistic meta-regression indicated that among the four domains, only the allocation sequence generation and the blinding were associated with the statistical significance of the outcomes. Studies that used correct sequences for generating the allocation presented a chance of presenting significant results that was 2.20 times greater, but the association was not significant (P = 0.07). Studies that used blinding reduced the chance of presenting significant results by a factor of 2.28 times.

## DISCUSSION

One of the limitations of this study was that it was restricted to evaluating the journals containing open-access studies that are indexed in Lilacs. These only represent part of the scientific production of a country or region. Some articles within the field of dentistry are published in journals with restricted access or in medical journals, and there is a growing tendency towards publication in dentistry in international journals that are not indexed in Lilacs.^88^ On the other hand, the present investigation used a hand search, which is considered to be the gold standard: it is more sensitive and retrieves between 92% and 100% of the RCTs. Traditional electronic searches may retrieve 42% of the RCTs, while searches using highly sensitive strategies (HSS) that do not exist for Lilacs may retrieve up to 80% of the RCTs.^89^ In the present study, the hand search retrieved 4,879 open-access studies. The quantity of open-access studies within dentistry went up from 2.44% in 2002 to 5.18% in 2007, in relation to the total number of health studies, independent of the subject.

The reason for the use of regional databases is the ability to complement the large international databases to adequately answer clinical questions and provide contextual evidence to support decision-making processes to meet the needs of local or regional populations and help solving the global information poverty.^90^ Clark and Castro^91^ analyzed the Lilacs database and found that in 71% of the cases, a search in this database could be useful for the authors of systematic reviews. The data from the present study indicated that, if evaluated systematically and rigorously, only 78 (1.70%) out of the 4,581 studies were potential RCTs and 10 (0.02%) were true RCTs, because only these 10 studies used correct allocation sequence generation.

The main role of correct random sequence generation for the allocation is to ensure that the groups are balanced, in relation to both known and unknown factors, thus equalizing the initial prognosis before introducing the experimental intervention. For this dimension, among the 78 RCTs evaluated, 64 (82.05%) did not report on it or used an incorrect method, four studies (5.13%) were classified as unclear and only 10 studies (12.82%) used a method that was truly randomized for generating the allocation sequence for the groups. Among these ten randomized studies, five of them used simple or unrestricted randomization, among which four studies^24,39,44,58^ used random number tables and one study^48^ used a computer-generated random sequence. The other five authors used restricted sequences for generating the allocation: Botelho et al.^17^ used randomization in blocks, from a computer-generated list; Proano de Casalino and Guillen Galarza,^65^ de Assis et al.^32^ and Peruzzo et al.^63^ tossed a coin; and Mora Loya et al.^55^ used a random number table, but in a restricted manner. Many authors reported that their study was randomized but did not report the method used and many mentioned randomization only in the title or abstract but did not report it in the text of the article. Other studies reported the randomization as synonymous with allocation to groups through the judgment of an experienced researcher.

Since 1995, there has been good empirical evidence regarding the effects from non-randomized allocation sequences. Schulz et al.^5^ found that, on average, studies with inadequate allocation sequences exaggerated the odds ratios by 41% and by 30% for unclearly concealed trials (after adjustment for other aspects of quality), in comparison with the studies that used adequate sequence allocation. The results suggested that if the allocation is not randomized, even in studies with correct allocation sequence concealment, it is possible for the investigators and patients to decipher the allocation of the participants to some degree. Recently Kunz et al.^92^ confirmed empirically that not using randomized generation of sequence allocation, or using it inadequately, could result in overestimates of the interventions. However, this bias may occur in either direction: in favor of or against the intervention. In the present review, correct use of sequence generation for the allocation doubled the chance that the study would furnish significant results, although the meta-regression did not produce a significant result. This was probably because of type II error due to the small sample of articles and the type of outcome used (statistical significance), which requires a major difference in effect size for it to be greater than what might be expected by chance.

The first meta-epidemiological study to provide empirical evidence regarding the effect from allocation concealment was published in 1995.^4^ This evidence was confirmed subsequently by other studies.^93,94^ These studies together involved evaluations on 1,615 primary studies. More recently, Wood et al.^95^ evaluated 102 meta-analyses and 804 studies and concluded that lack of allocation sequence concealment produced a mean increase in the estimates of 17%. This deviation did not occur or was smaller in studies that measured objective outcomes (9%) and was greater in subjective studies (31%). The importance of allocation sequence concealment is that its absence or incorrect use may result in bias in selecting participants based on prognostic factors, even in the presence of a correct method for generating the allocation. For example, when the result from the random draw is known, patients allocated to a given type of intervention may be considered inadequate and be eliminated from the study. Randomization associated with allocation sequence concealment may avoid selection bias, but when used alone, it may be insufficient to avoid this type of bias. In the present evaluation, 67 studies (85.90%) presented high risk of bias, four (5.13%) studies were unclear and only seven studies (8.97%) reported a correct method for the concealment of allocation. Among the latter, only two studies^58,63^ used complete control over selection bias, through concomitantly using randomization and allocation sequence concealment. Lima et al.,^50^ Ozaki et al.,^58^ Salgado et al.,^73^ Bortolluzy et al.,^16^ Costa et al.,^28^ Moraes et al.^56^ and Peruzzo et al.^63^ used central randomization by third parties, or sequentially numbered envelopes or packages containing medications or the test and control substances. Guimarães et al.^47^ used opaque sealed envelopes. Allocation sequence concealment was the least frequently observed dimension among the articles evaluated, and was the most frequent source of confusion with blinding.

Lack of blinding may lead to performance bias, thus signifying a systematic difference between the groups, with regard to the care received, because of intentional exposure to different co-interventions other than the experimental intervention. It may also induce detection bias, which results in systematic differences between groups regarding the way in which outcomes are measured or reported.^2^ While there is empirical evidence that generation and allocation sequence concealment are more homogenous regarding modification of the effect size of interventions, double blinding produces bias of smaller magnitude and the deviations are more heterogenous. Colditz et al.^96^ evaluated 113 RCTs and found that if correct blinding was used, the effects obtained from the interventions were more modest. Empirical studies have indicated that incorrect blinding increases the size of the effect from interventions by an average of 9%.^97^ The impact from lack of blinding varies according to the type of outcome. Objective and very marked outcomes such as mortality are less influenced by absence of blinding and sometimes may not need this type of control. Subjective studies are much more susceptible to this type of bias.^95^ In the present study, blinding was the bias control dimension most frequently seen among the studies evaluated. Out of the 78 studies, 26 (33.33%) used a correct method of blinding, even if not all of them were double blind. One study^46^ reported a triple blind method, while nine studies reported a double-blind method: de Assis et al.,^32^ Bortolluzy et al.,^16^ Botelho et al.,^17^ Calvo et al.,^19^ Faraco et al.,^36^ Guimarães et al.,^47^ Kogawa et al.,^48^ Rivera et al.^68^ and Vieira et al.^86^ Fifteen studies reported single blinding of the healthcare providers or outcome assessors.^10,23,25,27,28,30,31,33,37,39,44,46,50,58^ In another 14 studies, the authors reported blinding but did not describe the method, or blinding was impossible. The studies by Gispert Abreu et al.^41^ and Gispert Abreu et al.^42^ were examples of the impossibility of blinding, because there was no report of any alternative treatment or placebo for the controls.

In the present study, it was found that 23 studies (29.49%) were classified as presenting low risk of bias for the dimension of incomplete outcome data, while 55 studies (70.51%) presented an unclear or high risk of bias. Some studies stated that the number of participants at the end of the study was identical to the number that underwent the initial examination. For some of these studies, this coincidence did not seem plausible, which suggests that participants who started the study but did not complete it were simply disregarded, and that only the number of participants who completed the study was considered for the analysis. In such cases, the loss of participants was erroneously reported as zero. Among the studies that evaluated or reported missing data, none of them used intention-to-treat analysis.

The empirical evidence for evaluating the effect of incomplete outcome data on the magnitude of the effects from interventions was assessed by Tierney et al.^98^ They evaluated 14 meta-analyses on individual patients' data and found that incomplete outcome data had a real effect on the outcomes and that the bias was greater in the treatment group than in the control group. In 2009, Nüesch et al.^99^ evaluated 14 meta-analyses with subjective outcomes that included 167 trials and 41,170 participants. The results indicated that trials with incomplete outcome data tended to present results that were between 25% and 50% more favorable to the intervention.

Taking into account the set of 4,581 studies, only one study (0.22%) was classified as presenting low risk of bias for the four dimensions assessed and 77 potential RCTs (98.72%) were classified as presenting high risk of bias.

The level of bias control in the set of studies evaluated in Lilacs indicates that this is a body of evidence with less validity than that found in other databases such as Medline. When evaluating 3,631 studies on dental prostheses from journals indexed in Medline, Dumbrigue et al.^100^ found that 1.71% were RCTs and 16% had a low risk of bias. Montenegro et al.^101^ found that in periodontics, RCTs were frequently of poor quality. The sequence generation could only be assessed in 17% of the RCTs and the allocation was only concealed in 7% of the RCTs evaluated. Blinding was implemented slightly more frequently, in 55% of the RCTs. The dimension of incomplete outcome data could not be assessed in 44% of the RCTs. Sjögren and Halling et al.^102^ evaluated 200 medical and dental RCTs and found that, in relation to generation of randomization, although the RCTs mentioned the term "randomization", only one third of them presented a true method for generating randomization. Inadequate methods were reported in 6% of the RCTs and two thirds of the RCTs did not present any randomization method. Regarding allocation sequence concealment, these same authors found that only 13% of the dental RCTs and 9% of the medical RCTs reported adequate methods. With regard to blinding, they found that 28% of the dental RCTs and 4% of the medical RCTs implemented double blinding, but that the method was only adequate in 18% of the dental RCTs and 2% of the medical RCTs. Regarding incomplete outcome data, 35% of the dental RCTs and 44% of the medical RCTs reported this item, thus suggesting that the risk of bias due to incomplete data was low. In an assessment on RCTs relating to implant dentistry published over 10-year period (1991 to 2000), Dumbrigue et al.^100^ found 43 possible RCTs. The randomization method was explicit in 51% of the RCTs, but only 12% incorporated blinding in assessing the outcome. Looking at overall quality scores, only 2% of the RCTs adequately reported on control of bias in the three areas examined, while 56% were deficient in one area and 42% were deficient in two areas. Reporting of randomization procedures and blinding of outcomes for most of the implant RCTs was inadequate. Subject retention and documentation of subject withdrawals were adequately reported. Although the number of RCT's in dentistry with low risk of bias is not encouraging, the Lilacs database presents a worse situation than do other databases.

The distribution of the RCTs according to the importance that their authors gave to the issue of bias control, as reflected in the different degrees to which this was implemented, did not correspond to the potential for changing the effect size. This suggests that the same problem that exists in the gap between the best available clinical evidence and its adoption in practice^103^ is also an evident gap between the empirical evidence in meta-epidemiological studies and the choice of dimensions used in the studies. Guidelines and better dissemination of evidence relating to methodology are needed in order to reduce this gap.

The results from the present study indicated that the proportion of information coming from studies with a high risk of bias that were published in open access journals indexed in Lilacs was sufficient to seriously affect the interpretation of the results. The effective presence of bias seriously weakened the reliability of the results from the studies evaluated, thus indicating that there is practically no trustworthy evidence that clinicians and administrators can use as support for decision-making processes in the field of dentistry. In other words, the internal validity of most the RCTs evaluated was very poor. Future studies will need to investigate whether this is a question relating to the database examined (Lilacs) or to accessibility, given that all of the RCTs evaluated were available with open-access; or whether there is a situation of extreme insufficiency of quantity of and control for the risk of bias in RCTs within the field of clinical dental research in Latin America and the Caribbean region.

## CONCLUSION

Out of the 4,879 articles classified, only 10 studies (0.22%) were considered to be true RCTs and, of these, only a single study was classified as presenting low risk of bias.

All of the studies that controlled for three or more dimensions of potential sources of risk of bias presented non-significant results. The item that the authors of these RCTs most frequently controlled for was blinding. There is an evident gap between the empirical evidence produced by meta-epidemiological studies and the importance and frequency of use of bias control in studies on dentistry.

The effective presence of bias seriously weakened the reliability of the results from the dental open-access studies evaluated in the Lilacs database, such that they were of little use in dentistry for elaborating guidelines or systematic reviews as support for decision-making processes.
